# Endoplasmic reticulum protein 29 (ERp29), a protein related to sperm maturation is involved in sperm-oocyte fusion in mouse

**DOI:** 10.1186/1477-7827-8-10

**Published:** 2010-02-04

**Authors:** Xiaoqian Ying, Yue Liu, Qiangsu Guo, Fei Qu, Wei Guo, Yemin Zhu, Zhide Ding

**Affiliations:** 1 Shanghai Key Laboratory for Reproductive Medicine, Department of Histology and Embryology, School of Medicine, Shanghai Jiao Tong University, Shanghai 200025, China

## Abstract

**Background:**

Sperm-oocyte fusion is a critical step in fertilization, which requires a series of proteins from both spermatozoa and oocyte to mediate membrane adhesion and subsequent fusion. A rat spermatozoa membrane protein is endoplasmic reticulum protein 29 (ERp29), which significantly increases on the sperm surface as well as in the cytoplasm of epididymal epithelia from caput to cauda as the sperm undergo epididymal maturation. Moreover, ERp29 facilitates viral infection via mediating membrane penetration. We determined if in addition to promoting sperm maturation ERp29 may also play a role in facilitating gamete fusion during the fertilization process.

**Methods:**

Laser scanning confocal microscopy (LSCM) and Western blot analysis were employed to probe for ERp29 protein in BALB/c mouse epididymal and acrosome-reacted spermatozoa. We prepared rabbit polyclonal antibodies against mouse recombinant ERp29 (rERp29) to characterize: 1) fertilization rate (FR); 2) fertilization index (FI); 3) sperm motility and 4) acrosome reaction (AR).

**Results:**

Confocal microscopy indicated that ERp29 was partially localized at the sperm head of the epididymal caput as well as over the whole head and part of the principal piece of the tail region from the epididymal cauda. However, when the acrosome reacted, ERp29 remained in the equatorial and post-acrosomal regions of the sperm head, which is the initial site of sperm-oocyte membrane fusion. Such localization changes were confirmed based on the results of Western blot analysis. Furthermore, the antibodies against mouse rERp29 inhibited the spermatozoa from penetrating into the zona pellucida (ZP)-free oocytes. The functional blocking antibodies reduced both mouse sperm-oocyte FR and FI at concentrations of 100 and 200 micro g/ml compared with pre-immunized rabbit IgG or with anti-mouse recombinant bactericidal/permeability-increasing protein (BPI, a sperm surface protein unrelated to sperm-oocyte fusion) antibodies (100 micro g/ml), but they had no effect on sperm motility and AR.

**Conclusion:**

This study demonstrates that ERp29 on mouse spermatozoa membrane changes during epididymal transit and AR. Accordingly, in mice this protein may be one of the important factors involved in sperm fertilization by facilitating sperm-oocyte membrane fusion.

## Background

Sperm-egg plasma membrane interaction referred to as gamete fusion, is an extremely important step needed for mammalian fertilization [1]. In the past ten years, the mechanism of sperm-oocyte fusion has been extensively studied. The results of gene deletion analysis and the ZP-free oocyte insemination test suggested that several sperm membrane proteins were involved in this process, e.g., a disintegrin and metalloproteinase gene family members (ADAM) [2-4], cysteine-rich secretory protein (CRISP) [5,6] and Izumo [7]. Although these proteins appear to play a role in sperm-oocyte fusion, it is evident that there are many other unidentified essential proteins participating in this process.

Endoplasmic reticulum proteins (ERp), some of which are in the protein disulfide isomerase (PDI) family, play vital roles in protein secretion. In some cases, they are recognized primarily as catalysts of disulphide bond formation and chaperones of protein folding [8]. Recent reports showed that a 57 kDa ERp, ERp57, participate in sperm-oocyte fusion and is intimately associated with providing human sperm fertilization capability [9,10]. However, only a few of the described ERps have been identified in mammal spermatozoa or reported to be involved in spermatozoa fertility.

In our previous study, we first reported ERp29 in rat spermatozoa and confirmed that ERp29 was increased significantly on the sperm surface as well as in the cytoplasm of epididymal epithelia from caput to cauda during sperm epididymal maturation [11]. Furthermore, ERp29 could facilitate polyomavirus to infect a host cell via triggering a conformational change in virus and stimulating membrane binding [12]. We undertook the present study to verify ERp29 expression on BALB/c mouse sperm during epididymal transit and acrosome reaction, and further investigated the ERp29's functional role in sperm-oocyte fusion.

## Methods

### Chemicals and animals

Unless otherwise stated, all reagents were purchased from Sigma-Aldrich Corporation (St. Louis, MO, USA). Animal experiments were conducted according to the International Guiding Principles for Biomedical Research Involving Animals, as promulgated by the Society for the Study of Reproduction. White New Zealand rabbits (male: ~6 months old, body weight ~2.5 kg) and BALB/c mice (male: aged 9-11 weeks, body weight 22-24 g; female: aged 7-9 weeks, body weight 18-20 g) were purchased from Shanghai SLAC Laboratory Animal Corporation (Jiu-Ting, Shanghai, China) and accommodated in the animal facility for at least for 1 week prior to experimentation.

### Preparation of epididymal spermatozoa, sperm protein and membrane protein

Isolation of mouse epididymal caput and caudal spermatozoa, and protein extraction were performed as described [11]. Sperm membrane protein was isolated with the ReadyPrep Protein Extraction Kit (Pierce, Rockford, IL, USA). Protein concentration was determined by BCA™ Protein Assay Kit (Pierce), using bovine serum albumin (BSA) as a protein standard.

### Western blot analysis

Protein samples (20 μg per well) were separated under denaturing conditions using SDS-PAGE, and then transferred to polyvinylidene difluoride (PVDF) membranes (GE Healthcare, Waukesha, WI, USA) using a semi-dry transfer apparatus (Bio-Rad, Hercules, CA, USA). Membranes were blocked for 1 h at room temperature with Tris-buffered saline (TBS) containing 0.1% Tween-20 and 5% BSA. Immunoblotting was performed with rabbit polyclonal ERp29 antibody (Abcam, Cambridge, MA, USA) at a 1:1000 dilution, followed by incubation with secondary antibody conjugated to HRP (Abgent, San Diego, CA, USA) at a 1:5000 dilution. The blotting signals were detected by enhanced chemiluminescence (ECL) (ECL Plus, GE Healthcare), following the manufacturers' protocol. In the meantime, β-actin served as the internal control. Western blot analysis was repeated three times and then the results were scanned by Calibrated Densitometer (Bio-Rad GS-800). Finally, the averages were calculated with Quantity One (Version 4.6.1) software.

### ERp29 immunofluorescent staining

Mouse caput and caudal spermatozoa were isolated and immediately smeared onto individual slides. ERp29 immunofluorescent staining was performed as described [11]. Slides were blocked with 5% BSA and incubated with rabbit anti-mouse ERp29 antibodies (Abcam) or normal rabbit IgG (Upstate, Temecula, CA, USA) (1:500 dilution each). Goat anti-rabbit antibody linked with fluorescein isothiocyanate (FITC) (Rockland, Gilbertsville, PA, USA) was used as the secondary antibody at 1:300 dilution. Finally, the fluorescent-stained sperm samples were viewed under LSCM (Carl Zeiss LSM-510, Jena, Germany) and the graphs were processed with its accompanying AIM (Release Version 4.0 SP2) software. Indirect immunofluorescence analysis was repeated at least three times.

### Prokaryotic expression and purification of recombinant ERp29

Total RNA was extracted from mouse liver with RNA Easy (Qiagen, Valencia, CA, USA), according to the manufacturer's protocol. Then, RNA was transformed into cDNA with RT-PCR. A segment of ERp29 was amplified with the forward primer 5'-GC**GGATCC**TTCTACAAGGTCATTCCC-3'(containing BamH I site, bolded) and the reverse primer 5'-GC**AAGCTT**TAGCCCACTTCTTCTCTG-3'(containing Hind III site, bolded). Prokaryotic expression and purification of rERp29 were performed as previously described [13]. The new recombinant prokaryotic expression vector was named pET-28a(+)/ERp29, and propagated in *E. coli *BL21 (DE3) host cells cultured with 0.8 mM isopropyl-b-Dthiogalactopyranoside (IPTG) for 12 hours at 37°C with gentle shaking. The purified protein was freeze-dried in a Heto Freeze Dryer (Thermo Electron Corporation, Waltham, MA, USA), and its identity was confirmed with 15% SDS-PAGE analysis and Western blot using anti-His monoclonal antibody (Abgent).

### Production of polyclonal rabbit anti-rERp29 antibodies

Production of polyclonal rabbit anti-mouse rERp29 antibodies was performed as described [13]. The titer of the antiserum was measured using an indirect ELISA at 450 nm in conjunction with an Anthos Zenyth 1100 multimode detector (Anthos Labtec Instruments GmbH, Wals, Austria) with a 5-s pre-read shaker. Finally, the immunized rabbit IgG (including anti-rERp29 IgG) was purified through immunoaffinity chromatography from crude rabbit sera, using the ImmunoPure (G) IgG Purification kit (Pierce). In the meantime, normal rabbit IgG was purified from pre-immunized rabbit sera.

### Gametes preparation

Female BALB/c mice were superovulated by abdominal injection of 10 IU of pregnant mare's serum gonadotropin (PMSG; PROSPEC, Rehovot, Israel), followed 46-48 hours later by 10 IU of human chorionic gonadotropin (hCG; Li Zhu drug plant, Zhuhai, China). Cumulus enclosed-egg complexes were collected from the oviducts 15-16 hours after post-hCG injection, and treated with 0.1% hyaluronidase in Medium 16 (M16) to disperse the cumulus cells. The ZP was removed with 0.6% proteinase K, and then the eggs were incubated with M16 containing 3% BSA at 37°C, 5% CO_2 _for 30-60 minutes.

Mature spermatozoa were collected from epididymal cauda of BALB/c male mice by rough cutting in 1.5 ml of M16 containing 3% BSA; Motile spermatozoa were allowed to swim out for 10-15 minutes, and then the tissue was removed from the medium. One part of spermatozoa were cultured for 2 hours in M16 (containing 3 mg/ml BSA and 5 μmol/l ionophore A23187) at 37°C and 5% CO_2_, then used for localization of ERp29 in acrosome-reacted spermatozoa. The resting spermatozoa were cultured for 90 minutes in M16 containing 3 mg/ml BSA at 37°C, 5% CO_2_, allowing them to capacitate and undergo spontaneous AR. Following that, spermatozoa (adjusted to 10^7 ^sperm/ml) were incubated in the presence or absence of prepared and purified rabbit anti-mouse rERp29 antibodies (without sodium azide) or pre-immunized rabbit IgG (normal rabbit IgG) at different concentrations (20, 50, 100, 200 μg/ml), or rabbit anti-mouse rBPI antibodies (100 μg/ml, the antibodies were a generous gift from Dr Zhongping Zhou form at the Shanghai Key Laboratory for Reproductive Medicine, Shanghai, China), or washing buffer (control) for 40 minutes, and then diluted (1: 40) with M16 containing 3% BSA for use in the sperm-oocyte fusion assay and the final sperm concentration was 2.5 × 10^5 ^sperm/ml.

### Sperm-oocyte fusion assay

For every sperm-oocyte fusion assay, all the oocytes and spermatozoa used in an experiment were combined from several female and male mice and then divided into each group randomly. ZP-free oocytes were added directly to the sperm suspensions prepared as described above, and the gametes were co-incubated for 3 hours at 37°C, 5% CO_2_. Then, loosely bound spermatozoa were removed from the oocytes by gentle pipetting. After that, the oocytes were fixed with 4% paraformaldehyde for 15 minutes, and then treated with Hoechst 33342 for 10 min to stain the chromatin. Finally, images were collected using the LSCM and results were evaluated as the percentage of oocytes penetrated by sperm (FR) and the average number of penetrated sperm per oocyte (FI). Each assay was repeated at least three times at each concentration of IgG used in every experiment.

### Assessment of sperm motility and acrosome reaction

Spermatozoa from epididymal cauda were cultured for 90 minutes in M16 containing 3 mg/ml BSA at 37°C and 5% CO_2_, and then incubated either with prepared anti-mouse rERp29 antibodies or with normal rabbit IgG at the concentrations of 20, 50, 100, 200 μg/ml for 40 minutes. After sperm/antibody incubation, sperm motility (including movement parameters: velocity average path (VAP), velocity curvilinear (VCL) and velocity straight line (VSL)) was assessed using computer-assisted semen analysis (CASA; Hamilton-Thorn Research, Beverly, MA, USA) with parameters optimized for detection of mouse sperm.

AR assays were conducted essentially as described [14]. Sperm suspensions were fixed with 4% paraformaldehyde solution (pH 7.4) for 10 minutes, and then centrifuged and washed twice with 100 mM ammonium acetate (pH 9.0). The final sperm pellet was resuspended in 100 mM ammonium acetate, and 50 μl of the sperm suspension was smeared on a glass slide and incubated with Coomassie Blue (0.22% Coomassie Blue G-250, 50% methanol, 10% glacial acetic acid, 40% water) for 2 minutes. Finally, the percentage of acrosome-reacted cells was calculated by evaluating at least 200 spermatozoa and every experiment was repeated three times.

### Statistical analysis

Data are presented as mean ± SEM and analyzed using the SAS 8.2 statistical software. Fertilization rate was analyzed by chi-square test. Student-Newman-Kuel's test was performed to assess the significance of individual variations among the treated groups. Differences were considered statistically significantly different when *P *< 0.05.

## Results

### Identification and localization of ERp29 on spermatozoa

**Figure 1:**
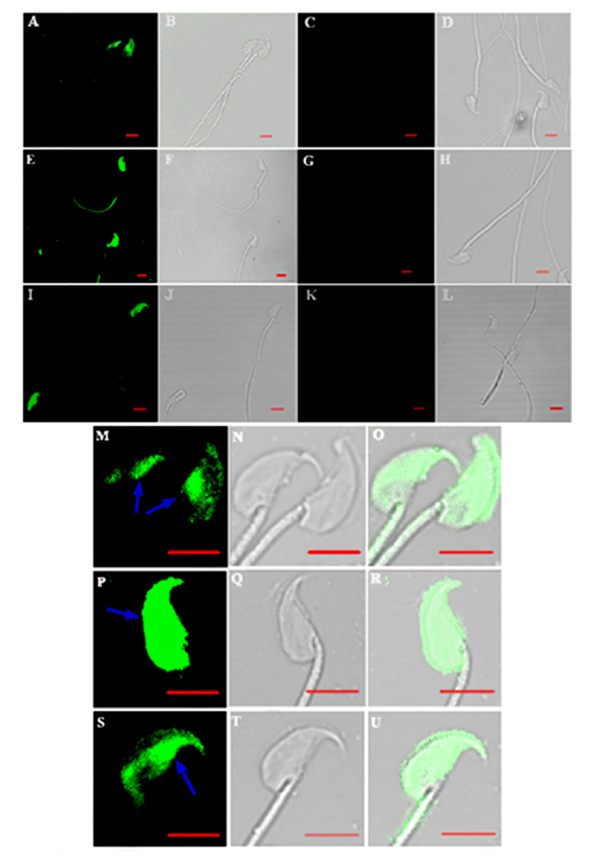
**Localization of ERp29 on BALB/c mouse spermatozoa by indirect immunofluorescence analysis**. (A), (E), (I) The localization of ERp29 on spermatozoa from epididymal caput, caudal regions and on acrosome-reacted spermatozoa. (B), (F), (J) Differential interference contrast (DIC) images corresponding to (A), (E), (I). (C),(G),(K) Caput, caudal and acrosome-reacted spermatozoa incubated with normal rabbit IgG as negative controls. (D), (H), (L) DIC images corresponding to (C), (G), (K). (M),(N) Caput sperm head in (A),(B) were magnified to show the precise localization. (P),(Q) Caudal sperm head in (E),(F) were magnified to show the precise localization. (S),(T) Acrosome-reacted sperm head in (I),(J) were magnified to show the precise localization. (O),(R),(U) The combined image of (M) and (N), (P) and (Q), (S) and (T), respectively. The arrows indicated the exact localization of ERp29 in the spermatozoa on different conditions. Calibration bar = 5 μm.

ERp29 was present in mouse spermatozoa from both epididymal caput and cauda. As indirect immunofluorescence showed, ERp29 was mostly localized on part of the spermatozoa head from the epididymal caput, whereas it was also localized on the whole head and in the tail region (principal piece) of spermatozoa from the cauda. Moreover, ERp29 was still present in mouse acrosome-reacted spermatozoa, mostly on the equatorial and post-acrosomal regions of sperm head, which are generally considered as the initial sites of sperm-egg fusion (Fig 1).

**Figure 2:**
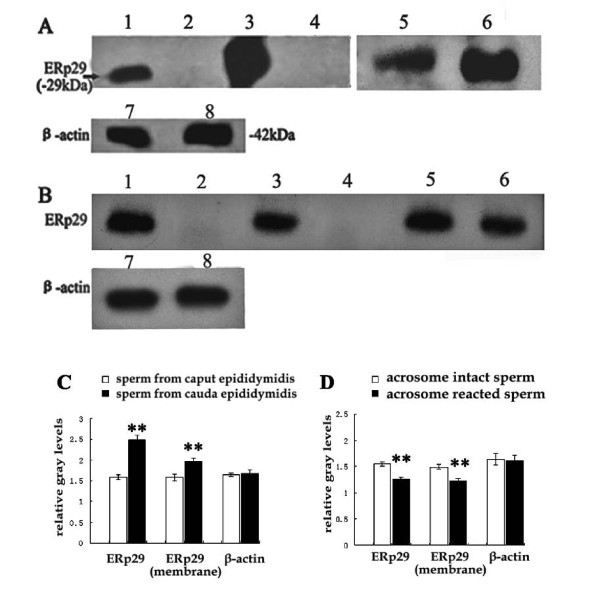
**Western blot analysis of ERp29 in BALB/c mouse epididymal and acrosome-reacted sperm**. (A) Lane 1, ERp29 in the whole protein from caput sperm; Lane 3, ERp29 in the whole protein from caudal sperm; Lane 5, ERp29 in the membrane protein from caput sperm; Lane 6, ERp29 in the membrane protein from caudal sperm; Lane 2 and 4, Normal rabbit IgG as negative control; Lanes 7 and 8, β-actin in the whole protein from caput and caudal sperm served as loading control. (B) Lane 1, ERp29 in the whole protein from acrosome intact sperm; Lane 3, ERp29 in the whole protein from acrosome-reacted sperm; Lane 5, ERp29 in the membrane protein from acrosome intact sperm; Lane 6, ERp29 in the membrane protein from acrosome-reacted sperm; Lane 2 and 4, Normal rabbit IgG as negative control; Lane 7 and 8, β-actin in the whole protein from acrosome intact and reacted sperm served as the loading control. (C) Average gray scale levels of the corresponding protein blot in (A) repeated three times. (D) Average gray scale levels of the corresponding protein blot in (B) repeated three times (**P < 0.01).

The results of Western blot analysis showed that the spot identified as ERp29, was significantly up-regulated in the sperm from caudal epididymis and then down-regulated in acrosome reacted sperm. Gel protein loading equivalence is documented by invariant levels of β-actin expression (Figs 2A and 2B). The ERp29 content in whole sperm protein as well as membrane protein revealed the same changes as found using indirect immunofluorescence analysis. This agreement confirmed that the ERp29 protein content was higher in the caudal sperm than in the caput or in acrosome-reacted sperm, especially on the sperm membrane (Figs 2C and 2D).

### Prokaryotic expression and purification of rERp29

**Figure 3:**
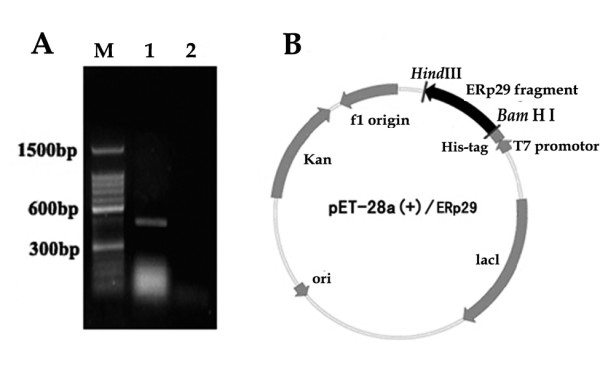
**Cloning the target fragment of ERp29**. (A) RT-PCR products of ERp29 fragment on 1% agarose gel. Lane M, marker; lane 1, ERp29 fragment; lane 2, negative control (PCR without cDNA). (B) Schematic of the recombinant plasmid pET-28a(+)/ERp29.

**Figure 4:**
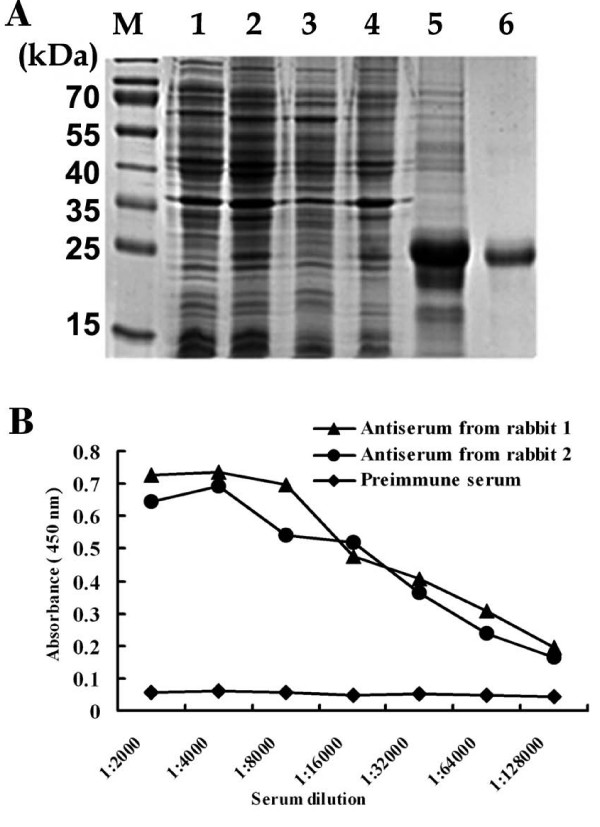
**Prokaryotic expression and purification of rERp29, and production of polyclonal anti-rERp29 antibodies in rabbits**. (A) Expression and purification of rERp29 fragment in *E. coli *BL21 (DE3) host cells. Different fractions of *E. coli *were separated on a Coomassie-blue-stained 15% SDS-PAGE gel. Lane M, protein molecular weight markers; lane 1, total cell lysates carrying pET-28a(+)/ERp29 fragment before IPTG induction; lane 2, total cellular protein after IPTG induction; lane 3, supernatant of cell lysate after ultrasonic treatment; lane 4, pellet of cell lysate after ultrasonic treatment; lane 5, protein purified based on its His6-tag by affinity chromatography employing a Ni^2+^-NTA His-binding resin; lane 6, protein eluted in PBS by cutting certain protein bands from the gel after preparative electrophoresis. (B) Values of ELISA absorbance at 450 nm using rERp29 protein immunized rabbit sera or pre-immune serum (control).

**Figure 5:**
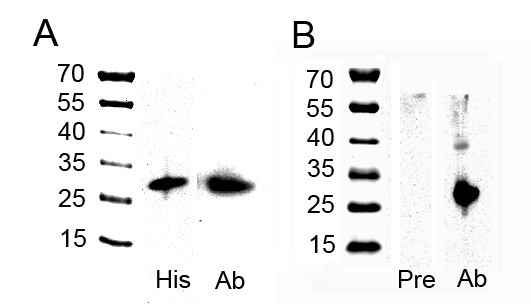
**The purified rERp29 protein was analyzed by Western blot**. (A) The purified rERp29 protein (~23 kDa) was confirmed by immunoblotting, using anti-His monoclonal antibody (1:1000 dilution) and rabbit anti-rERp29 IgG purified from sera (1:1000 dilution). (B) ERp29 (~29 kDa) was distinctly detected in BALB/c mouse sperm proteins using rabbit anti-rERp29 IgG (1:1000) but not with IgG from pre-immunized sera. His, anti-His monoclonal antibody; Ab, rabbit anti-rERp29 IgG purified from sera; Pre, IgG from pre-immunized sera.

To overexpress the rERp29 protein and raise antibodies, a protein fragment of good antigenicity was selected by DNAssist. A 463 bp fragment was amplified from Mus musculus cDNA by PCR and separated on a 1% agarose gel (Fig 3A). The target fragment of ERp29 was then cloned into the prokaryotic expression vector, which was named pET-28a(+)/ERp29 (Fig 3B). This construct is predicted to encode a 206 amino acid-containing recombinant protein with molecular weight of ~23 kDa. It was successfully overexpressed by inducing it with IPTG; this 23 kDa protein was absent in non-induced cells, shown on 15% SDS-PAGE gel (Fig 4A). The recombinant protein was purified and then confirmed by SDS-PAGE analysis (Fig 4A) and Western blot using anti-His monoclonal antibody (MAB) (Fig 5A).

### Production of polyclonal anti- rERp29 antibodies in rabbits

Polyclonal antibodies against the rERp29 protein were generated by immunization of white New Zealand rabbits. At day 35, rabbits were killed and sera were collected. Results from the indirect ELISA indicated that the titers of rabbit anti-rERp29 sera were fairly high compared with that of pre-immune serum (control), reaching 1:128000 (Fig 4B). Anti-rERp29 IgG (without sodium azide) was then purified from crude rabbit sera, confirmed by Western blot (Fig 5B) and stored at 4°C for functional analysis in vitro.

### Effects of ERp29 on sperm-oocyte fusion studied by rERp29 antibodies

**Figure 6:**
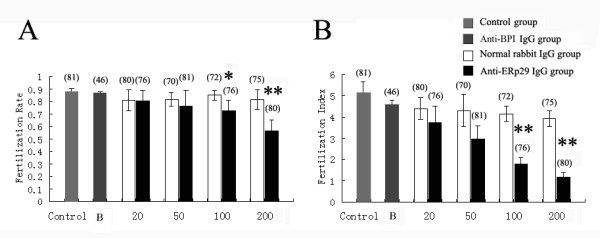
**Effect of anti-rERp29 antibodies on sperm penetration assay**. (A) FR at antibodies concentrations of 20, 50, 100, 200 μg/ml. (B) FI at the antibodies concentration of 20, 50, 100, 200 μg/ml (B was anti-rBPI at the antibodies concentration of 100 μg/ml; Data were presented as mean ± SEM. *P < 0.05, **P < 0.01). For all statistical figures, total number of oocytes used in each group was marked above the column.

To assess the possible role of ERp29 in mouse sperm-oocyte fusion during fertilization, we tested whether anti-rERp29 antibodies could inhibit mouse sperm from penetrating ZP-free oocytes. Thus, anti-mouse rERp29 antibodies (without sodium azide) generated from rERp29 immunized rabbits were used in these tests. Both the FR and FI were used to evaluate sperm ability to fertilize oocytes. Lower percentages of oocytes penetration were detected in anti-rERp29 antibodies treated sperm at the concentration of 100, 200 μg/ml (0.71 ± 0.08, 0.56 ± 0.09, respectively) compared with normal rabbit IgG (from pre-immunized sera) treated sperm (0.85 ± 0.04, 0.82 ± 0.08, respectively), or with anti-rBPI antibodies treated sperm (100 μg/ml, 0.87 ± 0.01), or with control sperm (0.88 ± 0.02) (Fig 6A, *P < 0.05, **P < 0.01; number of oocytes used in the experiments: Control = 81; Anti-rBPI = 46; Normal = 72, 75; Anti-rERp29 = 76, 80; respectively). Similarly, sperm fertilization index values were also significantly lower in anti-rERp29 antibodies treated sperm at concentrations of 100, 200 μg/ml (1.79 ± 0.30, 1.16 ± 0.22, respectively) compared with normal rabbit IgG treated sperm (4.13 ± 0.35, 3.91 ± 0.37, respectively), or with anti-rBPI antibodies treated sperm (100 μg/ml, 4.56 ± 0.25), or with control sperm (5.14 ± 0.51) (Fig 6B, **P < 0.01). In contrast to sperm, the anti-ERp29 antibodies had no significant effects on sperm-oocyte fusion when oocytes were pre-treated with the antibodies at a concentration of 100 μg/ml (*P *= 0.12, *P *= 0.34, for FR and FI respectively).

### Effects of ERp29 on sperm motility and AR studied by rERp29 antibodies

**Figure 7:**
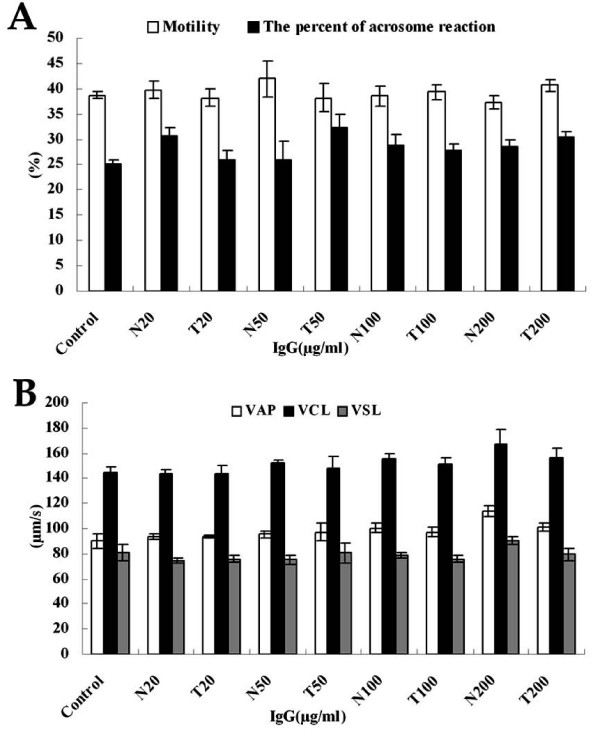
**Effect of anti-ERp29 antibodies on sperm motility and AR assay**. (A) Assessment of sperm motility and AR by different IgG treatments. (B) Assessment of sperm VAP, VCL and VSL by different IgG treatments (N: normal rabbit IgG; T: anti-rERp29 IgG; Values reported were mean ± SEM, *P *> 0.05).

To assess the possible role of ERp29 in mouse sperm motility and AR during fertilization, we tested whether anti-rERp29 antibodies could inhibit or facilitate sperm motility and AR. After sperm/antibody incubation at different concentrations, sperm motility (including movement parameters: VAP, VCL and VSL) was assessed using CASA. Sperm acrosome status was evaluated by Coomassie blue G250 stain. The results show that there are no significant differences between them. Both sperm motility and AR were unchanged in treated sperm at all anti-rERp29 antibody concentrations relative to those found in either normal IgG treated sperm, or blank control (P > 0.05) (Figs 7A and 7B). These results demonstrate that anti-rERp29 antibodies can significantly inhibit mouse sperm from penetrating the ZP-free oocytes. Such suppression was not attributable to changes in either the sperm motility or acrosome status.

## Discussion

ERp29 was first reported during proteomic investigations of mammalian tissues [15-17]. It is expressed ubiquitously in mammalian tissues [18-20] and the homologous proteins have been identified in organisms as primitive as the fruit fly [21]. As previously reported, ERp29 was widely thought to be an assistant for protein folding and has a probable function as a PDI like molecular chaperone. It may have a role in the production of endomembrane and secretory proteins, and also can bind to other ERps [19,20].

In our previous study, we found that ERp29 was apparently up-regulated as rat sperm underwent epididymal maturation and expressed mainly on caudal sperm [11]. We found in the current study that these changes also occur in BALB/c mouse. Our results indicate that in epididymal caput sperm, ERp29 was localized on the anterior region of head, whereas it was instead localized on the whole head and tail region (middle pieces) in sperm from cauda. Besides, ERp29 was still present in the equatorial and post-acrosomal regions of the sperm head after acrosome reacted, which are generally considered as the initial sites of sperm-egg fusion. This distribution of ERp29 suggests that it may be an important factor for mature sperm function, especially for fertilization processes, such as capacitation, AR and fusion with oocytes.

It is noteworthy recent studies report that ERp29 can facilitate virus membrane penetration or fusion into host cells [12,22]. Thus, we presumed that the expression pattern of ERp29 on mouse acrosome-reacted spermatozoa may also have a potential role in sperm fertilization, especially in sperm-oocyte membrane fusion. This presumption was tested by determining whether or not ERp29 present in the mouse spermatozoa membrane participates in sperm-oocyte fusion. Thus, we cloned the gene encoding mouse ERp29 and generated the relevant polyclonal antibodies. In our sperm penetration assay, we found that anti-mouse rERp29 antibodies could significantly reduce both mouse sperm-oocyte FR and FI at antibodies concentration of 100 and 200 μg/ml, but they had no effects on sperm motility and AR. In contrast to sperm, the anti-ERp29 antibodies had no significant effects on sperm-oocyte fusion when the oocytes were instead pre-treated with these antibodies. Furthermore, in order to remove any concern about nonspecific effects from anti-ERp29 antibodies, we employed anti-mouse rBPI antibodies as one kind of antibody control. BPI protein is one of the membrane proteins from mouse sperm head, which is not involved in sperm-oocyte membrane fusion [23]. The FR and FI results revealed that there is no significant difference between the preimmune IgG group (normal IgG) and antibodies against rBPI at an antibody concentration of 100 μg/ml, but a significant difference was observed between the anti-rBPI and anti-rERp29 groups. This strongly indicates that ERp29 may be involved in sperm fertilization by facilitating sperm-oocyte membrane fusion.

We addressed the question about how ERp29 affects sperm-oocyte membrane interaction. Until now there have no clear understanding of the molecular mechanisms underlying this phenomenon. Our results suggest that ERp29 on acrosome-reacted sperm membrane may play the same role as the PDI during sperm-oocyte fusion. Although PDIs have long been recognized as endoplasmic reticulum-resident proteins, recent studies have also found thiol isomerases on the surface of cells [24]. Gamete fusion may require a sperm surface-associated PDI, which can trigger a protein re-folding step leading to sperm-egg fusion. This mechanism relies on thiol-disulfide exchange, which may act in gamete fusion to produce conformational changes in fusion-active proteins [9]. PDI family members are characterized by the presence of a thioredoxin domain in their structures. Within this domain is the signature CxxC motif that allows the protein to cycle between a reduced and an oxidized state. In some cases, one of the Cys residues can be replaced by a different residue (e.g., CxxA). ERp29 has already been reported to be a member of PDI family [24] and contains a PDI-like structure: mature ERp29 (25.6 kDa, Mr 29,000 on SDS-PAGE) has an N-terminal domain homologous to the thioredoxin-like domains in PDI, and a C-terminal domain with similarities to the P5 subfamily of PDI [25-29]. Due to the fact that ERp29 contains only a single Cys residue in its entire sequence (Cys157), it remains either in the reduced state or forms a mixed disulfide bond with another endoplasmic reticulum factor. Thus, ERp29's reduced and mixed-disulfide bonded states may exhibit different conformations, enabling it to bind to substrates with different affinities. Alternatively, noncovalent interactions with other ER factors could change ERp29's conformation to drive the substrate binding and release cycle [12].

Besides, the mechanism of sperm-egg fusion regulated by ERp29 may also be presumed from the results of a study describing polyomavirus entrance into the endoplasmic reticulum of a host cell [22]. Down-regulation of PDI in cells is recently observed to inhibit polyomavirus infection and ERp29 was shown to trigger a conformational change in polyomavirus [30]. In this regard, ERp29 can alter the conformation of polyomavirus' coat protein VP1, internal protein VP2 and then stimulate polyomavirus to bind and perforate ER membrane [12,22]. The data suggest that ERp29 renders polyomavirus virions hydrophobic, allowing the entire viral particle to bind to the surface of the endoplasmic reticulum membrane; this step may prepare the virus for penetration across the lipid bilayer. In the meantime, dimerization of ERp29, mediated by N-terminal domain, serves as a general mechanism to regulate its endoplasmic reticulum activities [31]. Moreover, both ERp29 N-terminal and C-terminal domains are essential for inducing the local unfolding of polyomavirus to initiate the endoplasmic reticulum membrane penetration process [22,31,32]. Although the above explanations are all tenable based on our findings, there are additional questions that need to be addressed regarding the involvement of ERp29 in sperm-oocyte fusion. e.g., what is the relationship between Izumo and ERp29? Izumo is a critical factor involved in sperm-ooctye fusion localized on both mouse and human spermatozoa and detected exclusively in testis and sperm [7]. ERp29 and Izumo have poor sequence homology (only 33% identity in a 36 amino acids fragment, analyzed by protein-protein BLAST at NCBI). It remains unclear why both of them have effects on sperm-oocyte fusion, but have none on sperm AR.

## Conclusion

Our current studies initially demonstrated that ERp29 is present on mouse sperm membrane and is apparently up-regulated as the sperm undergoes epididymal maturation. It still remains in the equatorial and post-acrosomal regions of sperm head after AR. Anti-mouse rERp29 antibodies significantly reduced both mouse sperm-oocyte FR and FI, but they had no effect on sperm motility and AR. These results indicate that ERp29 has a potential function in mammalian fertilization and may be one of the novel factors in sperm-oocyte fusion.

## Competing interests

The authors declare that they have no competing interests.

## Authors' contributions

XY and YL participated in all aspects of the experiment. QG carried out the immunofluorescent staining. FQ, WG and YZ partially participated in the Western blots, antibodies generation and sperm-oocyte fusion analyses. ZD conceived of the study, and participated in its design and edited the manuscript. All authors read and approved the final manuscript.
